# Supplementary figures and images for: Gut transcriptome reveals differential gene expression and enriched pathways linked to immune activation in response to weaning in pigs
Source: Front Genet. 2022 Oct 24;13:961474. doi: 10.3389/fgene.2022.961474 (PMC9638111; doi:10.3389/fgene.2022.961474)

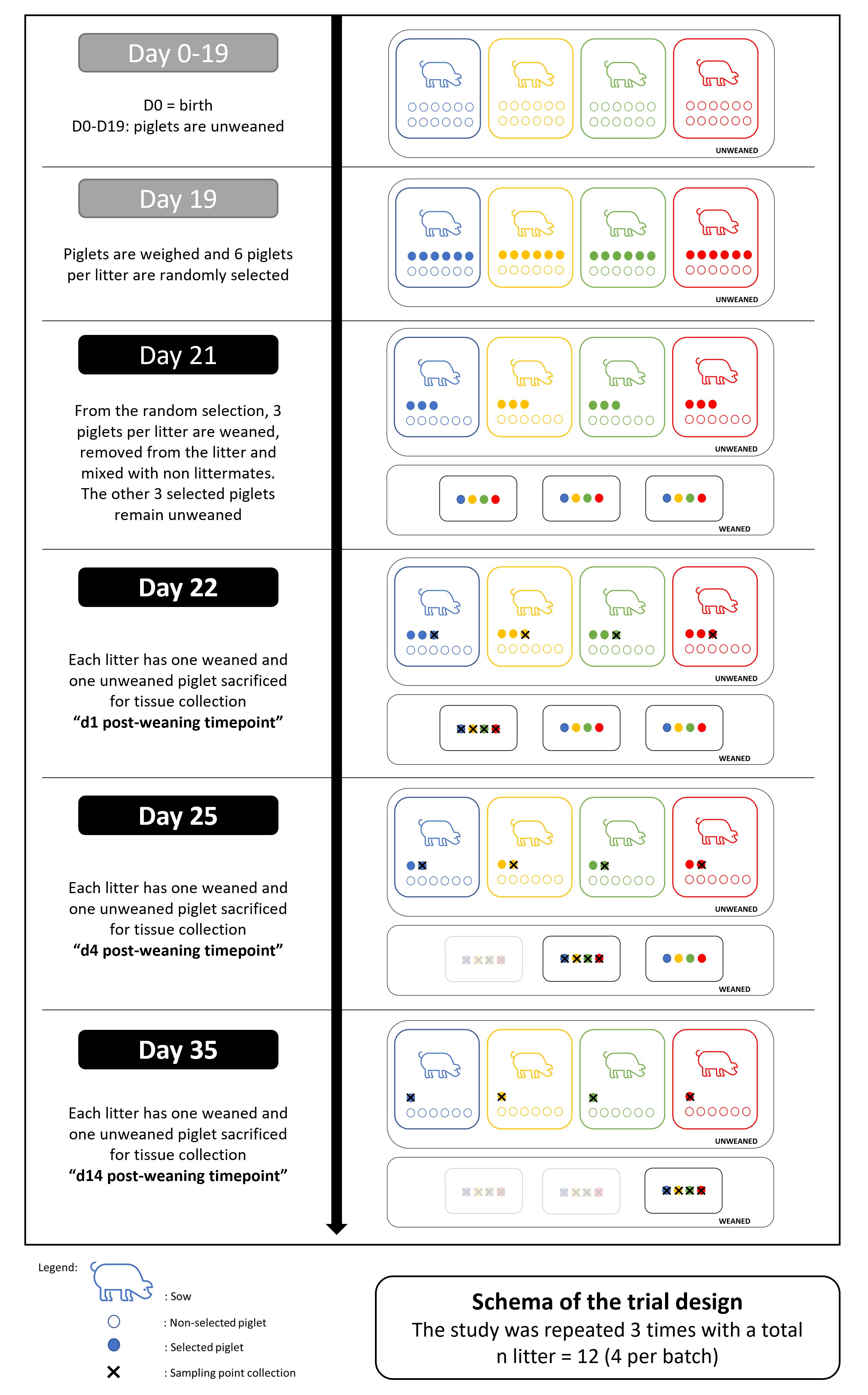

Supplement: Supplementary file 5 [file Image1.PNG]
